# Metabolomics profiling distinctively identified end-stage renal disease patients from chronic kidney disease patients

**DOI:** 10.1038/s41598-023-33377-8

**Published:** 2023-04-15

**Authors:** Lina A. Dahabiyeh, Refat M. Nimer, Khalid M. Sumaily, Mohamad S. Alabdaljabar, Minnie Jacob, Essa M. Sabi, Maged H. Hussein, Anas Abdel Rahman

**Affiliations:** 1 Division of Pharmaceutical Sciences, School of Pharmacy, The University of Jordan, Amman, 11942 Jordan; 2 Department of Medical Laboratory Sciences, Jordan University of Science and Technology, Irbid, 22110 Jordan; 3 Clinical Biochemistry Unit, Pathology Department, College of Medicine, King Saud University, Riyadh, 11461 Saudi Arabia; 4 Clinical Biochemistry Unit, Laboratory Medicine, King Saud University Medical City, King Saud University, Riyadh, 11461 Saudi Arabia; 5 Metabolomics Section, Department of Clinical Genomics, Center for Genomics Medicine, King Faisal Specialist Hospital and Research Centre (KFSHRC), Riyadh, 11211 Saudi Arabia; 6 Department of Internal Medicine, Mayo Clinic, Rochester, MN 55902 USA; 7 Department of Medicine, King Faisal Specialist Hospital and Research Centre (KFSHRC), Riyadh, 11211 Saudi Arabia; 8 Department of Biochemistry and Molecular Medicine, College of Medicine, Alfaisal University, Riyadh, 11533 Saudi Arabia

**Keywords:** Genetics, Biomarkers, Diseases, Endocrinology

## Abstract

Chronic kidney disease (CKD) is a serious public health problem characterized by progressive kidney function loss leading to end-stage renal disease (ESRD) that demands dialysis or kidney transplantation. Early detection can prevent or delay progression to ESRD. The study aimed to gain new insights into the perturbed biochemical reactions and to identify novel distinct biomarkers between ESRD and CKD. Serum samples of 32 patients with ESRD (n = 13) and CKD (n = 19) were analyzed using chemical isotope labeling liquid chromatography-mass spectrometry metabolomics approach. A total of 193 metabolites were significantly altered in ESRD compared to CKD and were mainly involved in aminoacyl-tRNA biosynthesis, branched-chain amino acid (BCAA) biosynthesis, taurine metabolism, and tryptophan metabolism. Three kynurenine derivatives, namely, 2-aminobenzoic acid, xanthurenic acid, and hydroxypicolinic acid were upregulated in ESRD compared to CKD due to the significant decrease in glomerular filtration rate with the progression of CKD to ESRD. *N*-Hydroxy-isoleucine, 2-aminobenzoic acid, and picolinic acid yielded AUC > 0.99 when analyzed using Receiver Operating Characteristic (ROC) analysis. Our findings suggest that inhibiting the kynurenine pathway might be a promising target to delay CKD progression and that metabolites with high discriminative ability might serve as potential prognostic biomarkers to monitor the progression of CKD to ESRD or used in combination with current markers to indicate the status of kidney damage better.

## Introduction

The kidneys play an important role in acid–base balance, plasma volume, serum osmolality maintenance, and hormone secretion, which are critical for maintaining human body homeostasis. However, these functions may be impaired in numerous kidney illnesses resulting in the loss of renal function^1^. Chronic kidney disease (CKD) is characterized by a progressive decrease in kidneys’ function that could eventually lead to end-stage renal disease (ESRD) that demands dialysis or kidney transplantation^2^. CKD is rapidly becoming a global public health problem^1^. Even though renal transplantation is the recommended treatment option for patients with ESRD, most patients are on dialysis while waiting for a kidney donor^3^. The worldwide cost of treating ESRD is expected to be more than $1 trillion^4^. Moreover, patients with ESRD have a 100 times higher death risk for cardiovascular diseases than general population^5^. A variety of different factors may cause CKD. The most typical reasons for developing CKD are diabetes mellitus and hypertension, whereas less common reasons include glomerulonephritis and polycystic kidney disease^6,7^.

Measurement of protein in the urine (proteinuria), which is considered a biomarker of kidney damage, and estimation of renal function (e.g., estimated Glomerular Filtration Rate, eGFR) are used in the regular clinical evaluation of CKD^2^. According to current guidelines from Kidney Disease Outcomes Quality Initiative (KDOQI), CKD is categorized based on the eGFR and urine albumin excretion rate (AER)^8^. There are five stages of CKD: stage 1 (≥ 90 mL/min/1.73 m^2^); stage 2, eGFR between 60 and 89 mL/min/1.73 m^2^; eGFR of 30–59 mL/min/1.73 m^2^ is considered stage 3; eGFR of 15–29 mL/min/1.73 m^2^ is considered stage 4; and eGFR less than 15 mL/min/1.73 m^2^ is considered stage 5^8^. Stages 1–2 of CKD show minor signs or symptoms, and the illness is often diagnosed only in its later stages^7^. Kidney failure is also called ESRD and it is considered stage 5 of CKD. The progression of CKD to ESRD is characterized by toxic waste builds up in the body, electrolyte imbalances and acid–base abnormalities, and dysfunction of certain endocrine functions resulting in various autotoxic symptoms^9,10^.

Current biomarkers used for kidney damage assessment have several limitations. For example, factors unrelated to the kidney (e.g., muscle mass or advanced age) might alter eGFR estimation using serum creatinine, which is linked with significant inaccuracy^11^. Similarly, fever and infection may influence the evaluation of proteinuria (albuminuria)^2^. Furthermore, in many primary renal disorders, proteinuria might continue even after the effective healing of kidney damage, resulting in needless therapy escalation for many patients^12,13^.

For the diagnosis of CKD, serum creatinine and blood urea nitrogen (BUN) are used. However, with a significant loss in renal function, there may be no change in serum creatinine concentrations, implying that kidney damage is already present or has started prior to the raise in serum creatinine level^1,14^. In addition, the sensitivity and specificity of serum creatinine and BUN for detecting renal damage are both low^15^. Progression of CKD to ESRD is associated with the accumulation of metabolites, toxic substances and uremic solutes in the plasma^1^. Therefore, identifying new biomarkers to increase the accuracy of the diagnosis and the assessment of CKD, and aid in the prognosis of ESRD progression is urgently required.

Metabolomics is a powerful analytical approach for investigating a group of small molecules (typically < 1500 Daltons) (like amino acids, lipids, and carbohydrates) called metabolites in cells, biofluids, tissues, and organisms^16^. Global metabolomics gives a comprehensive view of metabolisms, with implications for metabolic abnormalities that might explain disease pathogenesis^17,18^, and identify potential biomarkers^19–21^. Several studies used metabolomics to identify renal disease-related biomarkers to enable the early detection of CKD and distinguish its different stages^1,2,16,22,23^. Yet, limited literature is available on the use of metabolomics to identify potential biomarkers between CKD and its advanced state ESRD. Metabolomics was used to explore metabolic changes among ESRD patients with or without depression^24^ and to identify the metabolites with the ability to predict early kidney deterioration^25^. Early detection of renal damage will facilitate treatment and preventive measures which can delay or even prevent progression to ESRD^26,27^. Plasma metabolic alterations in ESRD have not yet been fully understood, and no studies have explored the metabolic differences between CKD and ESRD patients. Therefore, in this study, we used a chemical isotope labeling liquid chromatography-mass spectrometry (CIL LC–MS) metabolomics approach to identify novel distinct biomarkers between ESRD and CKD. The CIL LC–MS platform targets the amine/phenol sub-metabolomes (i.e., amino acids, nucleotides) that are involved in various central metabolism pathways. The identification of stage-specific metabolites significantly altered between CKD and ESRD may potentially improve the diagnosis and facilitate the treatment of CKD.

## Experimental section

### Subject recruitments

Samples of 32 patients were collected from the routine laboratory workup, after a regular visit to the department of medicine’s clinic at King Faisal Specialist Hospital and Research Center (KFSHRC) (Riyadh, Saudi Arabia). The study protocol was reviewed and approved by KFSHRC’ institutional research board (IRB) (approval number RAC 2160027). Patients were divided into two groups; ESRD (n = 13) and CKD (n = 19). In ESRD, 8 patients were on hemodialysis and 4 on peritoneal dialysis. Patients in CKD group had different stages of CKD with a mean eGFR of 46.74 mL/min and dipstick proteinuria ranging from 0 to 3, while protein/creatinine ratio (PCR) ranged between 2 and 1113 µmol/mg. A total of 32 healthy subjects with no medical history were enrolled from KFSHRC and served as controls. The healthy control subjects were selected from a biobank, built for our clinical metabolomics program, using an in-house developed algorithm for statistically matching the target disease cohort from Age, gender and others. This is our standard IRB approved practice for our clinical metabolomics program in rare disease. The demographic and clinical characteristics of the study groups are presented in Table 1.

**Table 1:** Demographic and clinical characteristics of CKD and ESRD study groups.

Demographic and clinical characteristics	CKD (n = 19)		ESRD (n = 13)	
	Mean	SD	Mean	SD
Age (years)	32.4	11.2	34.3	14.8
Gender^a^ (Male)	11	NA	4	NA
(Female)	8	NA	9	NA
BMI (kg/m^2^)	27.2	8.1	25.0	5.0
Systolic blood pressure (mmHg)	124.1	14.4	124.8	25.7
Diastolic blood pressure (mmHg)	74.4	16.0	71.0	11.2
EPI eGFR (mL/min)	46.7	29.8	< 15	NA
Serum creatinine (μmol/L)^b^	200.0	92.5	772.3	394.1
Urea (mmol/L)^b^	10.4	7.0	17.2	5.6
Protein dipstick	1.3	1.1	2.3	0.50
Urine protein/creatinine ratio (μmol/mg)	180.6	302.0	101.7	75.7

*BMI* body mass index, *eGFR* estimated glomerular filtration rate.

^a^Presented as the number of subjects in each group.

^b^Significantly different between CKD and ESRD (independent t-test, P value < 0.01).

### CIL LC–MS profiling on serum for CKD and ESRD patients

The separated serum samples were labeled by12C dansyl-chloride (DnsCl), while a pooled sample was generated by mixing all individual serum samples and labeled with 13C-DnsCl and prepared for chemical isotope labeling liquid chromatography-mass spectrometry (CIL LC–MS) as previously described^28^. The 13C-labeled pooled sample served as a reference for all the 12C-labeled individual samples. The pooled quality control (QC) samples were injected once every 15 LC–MS run, where peak pairs with ratio values having >  ± 25% RSD in the QC samples were filtered out. After normalization LC-UV quantitation was performed to determine the total concentration of dansyl-labeled metabolites. Thermo Fisher Scientific Dionex Ultimate 3000 UHPLC System (Sunnyvale, CA, USA) linked to a Bruker Maxis II quadrupole, time-of-flight (Q-TOF) mass spectrometer (Bruker, Billerica, MA) was used for sample analysis. An Agilent reversed-phase Eclipse plus C18 column (2.1 mm × 10 cm, 1.8 μm particle size, 95 Å pore size) was used as LC column, while the mobile phase A was 0.1% (v/v) formic acid in 5% (v/v) ACN, and solvent B was 0.1% (v/v) formic acid in acetonitrile. The LC gradient was: t = 0 min, 20% B; t = 3.5 min, 35% B; t = 18 min, 65% B; t = 21 min, 99% B; t = 34 min, 99% B, with a flow rate of 0.18 mL/min. The MS conditions were as follows: polarity, positive; dry temperature, 230 °C; dry gas, 8 L/min; capillary voltage, 4500 V; nebulizer, 1.0 bar; endplate offset, 500 V; spectra rate, 1.0 Hz.

### Data processing and statistical analysis

Acquired LC–MS data were processed by Bruker Daltonics Data Analysis 4.3 Software, UK. All data were aligned based on the peak’s accurate mass and retention time, and any missing values were filled by Zerofill software^29^. The raw data among the study groups were then subjected to uni- and multivariate analyses to analyze the differences in the metabolic profile between the groups. Data sets were normally distributed to the total sample median, log-transformed, and Pareto scaled. Multivariate analysis was carried out using SIMCAP + 14 (Umetrics AB, Sweden) to generate partial least squares-discriminant analysis (PLS-DA) and orthogonal PLS-DA (OPLS-DA) models. The robustness of the created models was evaluated by monitoring the fitness of model (R^2^) and predictive ability (Q^2^) values. Models that yielded large R^2^ (close to 1) and Q^2^ (> 0.5) values were considered appropriate models^30^. OPLS-DA models were also validated using a permutation test (100 permutations). Variable importance in the projection (VIP) value > 1 was used to identify significantly altered mass ions/metabolites between groups in OPLS-DA models^31^.

Univariate analysis was performed on MetaboAnalyst version 5.0 (McGill University, Montreal, Canada)^32,33^. Student’s independent t-test to identify significantly altered mass ions/metabolites among the two groups (CDK vs ESRD). A false discovery rate (FDR) less than 0.05 is defined as significant in our study groups. Volcano plots were generated applying FDR values less than 0.05 with fold changes (FC) cutoff of 1.5. Metabolites significantly altered in multivariate and univariate analyses were subjected to pathway analysis in MetaboAnalyst version 5.0 software.

### Metabolites identification

A three-tier ID approach was used to perform metabolite identification as described in our previous publications^34^. In Tier 1, metabolites were identified based on accurate mass, fragmentation ions, isotopic distribution, and retention time against a CIL library (Amino/Phenol sub-metabolome) that contains 711 experimental entries based on standard materials^35^. Then after metabolites were positively identified in tier 2 against linked identity library (LI Library) based on accurate mass and predicted retention time matches. In Tier 3 putative identification was performed by searching, accurate mass, against the MyCompoundID (MCID) library composed of 8021 known human endogenous metabolites (zero-reaction library) and their predicted metabolic products from one metabolic reaction (375,809 compounds, one-reaction library) and two metabolic reactions (10,583,901 compounds, two-reaction library)^36,37^.

## Results

### Demographic and clinical characteristics

Table 1 shows the characteristics of the study population. A total of 19 and 13 patients were included in CKD and ESRD groups, respectively, while 32 samples obtained from age and BMI matched healthy subjects served as controls. CKD and ESRD groups were matched with regard to age, body mass index (BMI), and blood pressure with no significant differences between the two groups. Patients in the CKD group had an eGFR of 46.74 ± 29.8, dipstick proteinuria ranging from 0 to 3, and protein/creatinine ratio ranging between 2 and 1113 µmol/mg. For ESRD, eGFR was within the category of renal failure range (< 15) based on the Kidney Disease Improving Global Outcomes (KDIGO) classification^38^.

Besides, patients in ESRD group had protein urea and protein/creatinine ratio of 2.3 ± 0.5 and 101.7 ± 75.7 which were not statistically significant compared to CDK group. Patients in ESRD had significantly higher level of serum creatinine and urea, Table 1.

### Mass ion detection and metabolites identification

A total of 5931 mass ion features were detected. Peak pairs without data present in at least 80.0% of samples in any group were filtered out to ensure data quality. After filtering, 4702 peak pairs were retained. Metabolites were identified using the Three-tier ID approach. In tier 1, 313 peak pairs were positively identified while 327 peak pairs were putatively identified in tier 2. The remaining peak pairs were identified in tier 3 and resulted in 678, 1702 and 862 peak pairs matching in the zero-, one- and two-reaction libraries, respectively (Supplementary Fig. S1). Thus, out of the 4702 unique peak pairs detected, 3882 pairs (82.6%) could be positively identified or putatively matched. Metabolites identified in tier 1 and tier 2, representing 13.6% of the total identified metabolites, are considered high confidence identification (mass error < 5.0 ppm) and can be used for pathway analysis and biomarker discovery.

### Overview of the three study groups (controls, CRD and ESRD)

**Figure 1:**
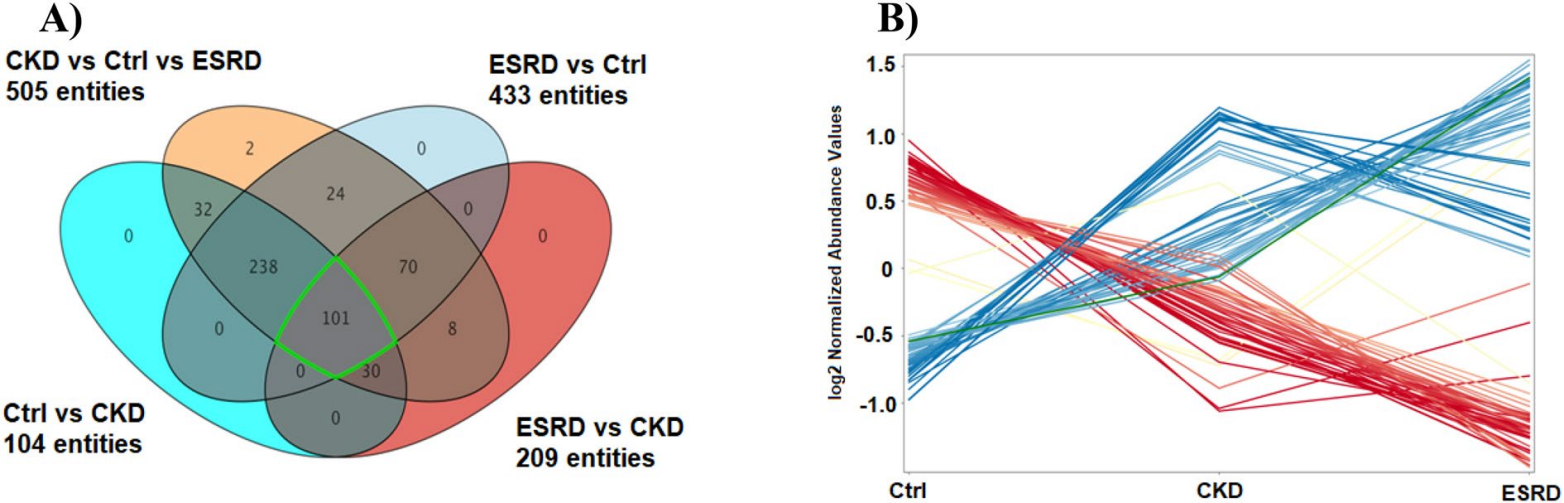
Statistical and fold change evaluation between the study groups control (Ctrl), chronic kidney disease (CKD), end stage renal disease (ESRD)). (**A**) Venn diagram showing One-way ANOVA between the study groups using Tueky’s Post-hoc analysis FDR P < 0.05. (**B**) 101 metabolic features were dysregulated significantly between the study groups.

**Figure 2:**
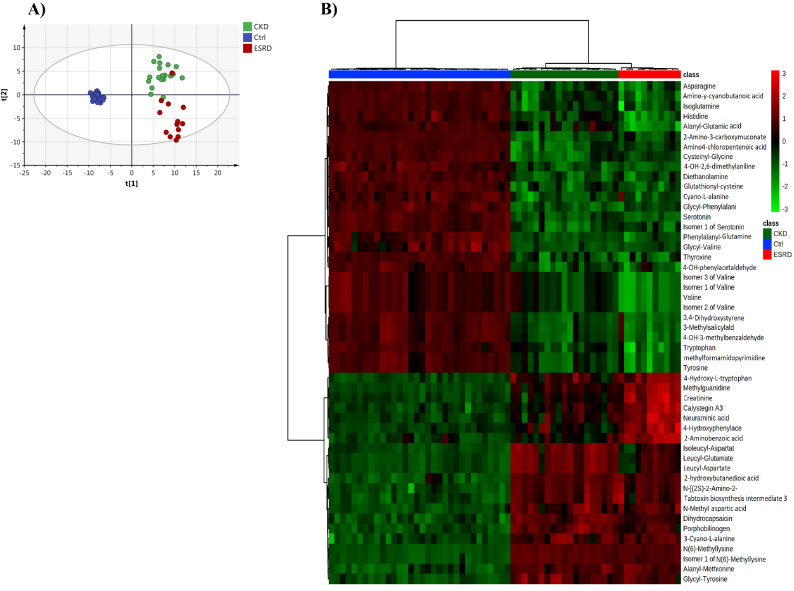
(**A**) PLS-DA scores plot (R2X: 0.43, R2Y:0.84, Q2 0.75) of the three study groups; controls (blue), ESRD (red) and CKD (green). (**B**) Hierarchal clustering (HAC) and heatmap analysis of the top 50 significantly altered metabolites between the three study groups; controls (blue), ESRD (red) and CKD (green). *ESRD* end-stage renal disease, *CKD* chronic kidney disease.

A Venn diagram was used to obtain an overview of the significantly altered ions between the groups (Fig. 1A). Additionally, the PLS-DA model was generated to examine any sample clustering and group separation in the data sets and identify any possible outliers (Fig. 2). Venn diagram revealed that 505 metabolites were dysregulated in the three data sets, of which 101 metabolites were common between the three groups (Fig. 1A,B). Expectedly, the largest change in metabolites level was detected between healthy controls and ESRD with 433 significantly altered metabolites.

PLS-DA scores plot revealed evident separation and clear clustering of the control group compared to CKD and ESRD (Fig. 2A). The latter two groups were separated from each other, however, one patient in the ESRD had a metabolic profile similar to CKD and thus was considered as an outlier and was not included in further analysis.

Visualization of the top perturbed metabolites between the three groups is presented in the heatmap in Fig. 2B. Significant changes in the level of tens of metabolites were noticed between the control group compared to ESRD and CKD. Serotonin, thyroxine and several amino acids including tyrosine and tryptophan were significantly decreased in CKD and ESRD compared to healthy controls. On the other hand, the level of creatinine and the uremic toxin methylguanidine was significantly higher in CKD and ESRD reflecting renal damage.

### Metabolic alterations in ESRD compared to CKD

**Figure 3:**
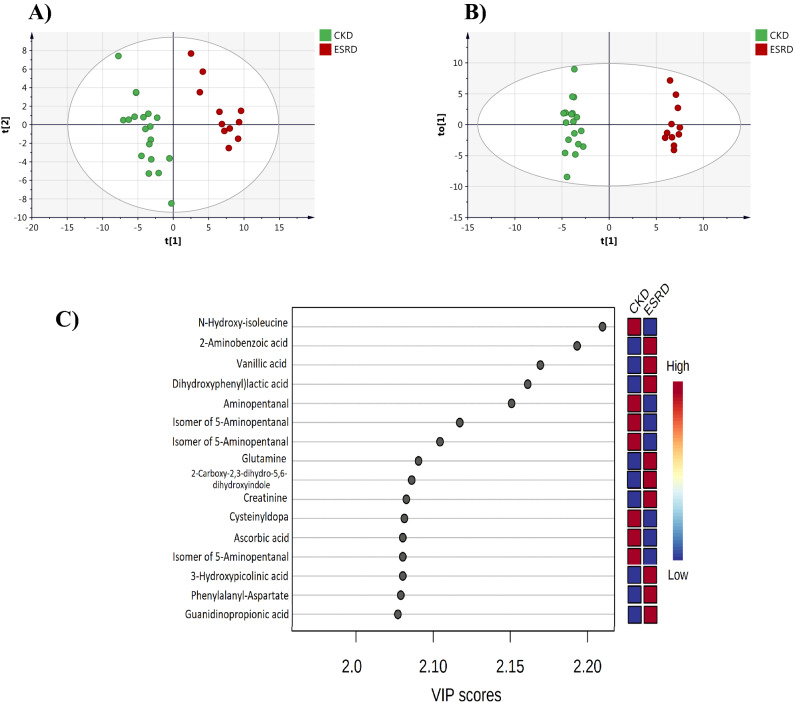
(**A**) PLS-DA scores plot (R2X: 0.54, R2Y:0.99, Q2 0.87). (**B**) OPLS-DA scores plot (R2X: 0.54, R2Y:0.99, Q2: 0.87) of CKD and ESRD groups. (**C**) Metabolites with the highest VIP scores in OPLS-DA model with their level in CKD and ESRD. *ESRD* end-stage renal disease, *CKD* chronic kidney disease.

The metabolic profile in ESRD was compared to CKD using multivariate and univariate analyses to extract significantly altered metabolites between the two disease states. In multivariate analysis, clear separation of the two groups was obtained with both PLS-DA and OPLS-DA models, Fig. 3A,B, respectively. The two models yielded satisfactory R2Y and Q2 values reflecting robust models with good predictive ability. OPLS-DA model passed the permutation validation test (Supplementary Fig. S2) and was used to identify metabolites with VIP > 1. A total of 217 metabolites were significantly different between ESRD and CKD and hence were responsible for the class separation in the OPLS-DA scores plot. *N*-Hydroxy-isoleucine, 2-amino benzoic acid and vanillic acid were associated with the highest VIP values as shown in Fig. 3C. The level of N-hydroxy-isoleucine was decreased in ESRD while 2-amino benzoic acid and vanillic acid levels were increased in ESRD compared to CKD (Fig. 3C).

**Figure 4:**
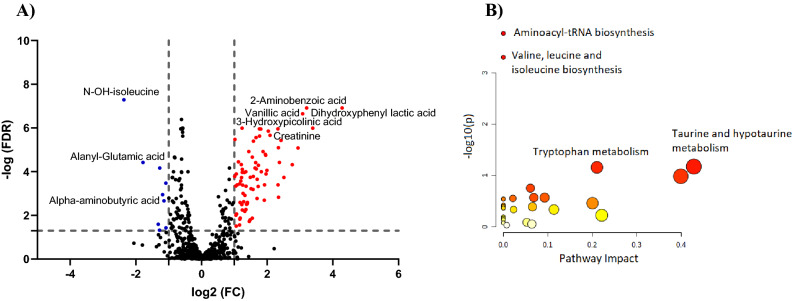
(**A**) Volcano plots of up (red) and down (blue) regulated metabolites in ESRD compared to CKD using FDR and FC cutoffs of < 0.05 and 2, respectively. (**B**) Summary of the most affected pathways. The node color and size are based on the p-value and the pathway impact value, respectively.

Univariate analysis using FDR < 0.05 revealed that 193 metabolites were altered between the two groups. Volcano plot applying FDR and fold change (FC) thresholds of 0.05 and 2, respectively, showed that 78 and 9 metabolites were significantly up- and down-regulated, respectively, in ESRD compared to CKD, Fig. 4A. Similar to multivariate analysis results, the level of vanillic acid, creatinine, and dihydroxyphenyl lactic acid was significantly increased in ESRD while the level of hydroxyl isoleucine and α-aminobutyric acid was decreased in ESRD compared to CKD (Fig. 4A).

### Potential biomarkers and pathway analysis

A total of 193 metabolites were significantly altered in ESRD compared to CKD in both univariate and multivariate analyses. The identity of these metabolites together with their FDR, VIP and FC values are presented in Supplementary Table S1. Pathway analysis revealed that aminoacyl-tRNA biosynthesis, valine, leucine and isoleucine biosynthesis, taurine metabolism and tryptophan metabolism are the most significantly altered pathways in ESRD compared to CRD, Fig. 4B. Among the significantly decreased metabolites in ESRD compared to CKD is branched chain amino acid (BCAAs; valine, leucine, and isoleucine) and taurine. On the other hand, three kynurine derivative metabolites; 2-aminobenzoic acid, xanthurenic acid and hydroxypicolinic acid were upregulated in ESRD compared to CRD.

**Figure 5:**
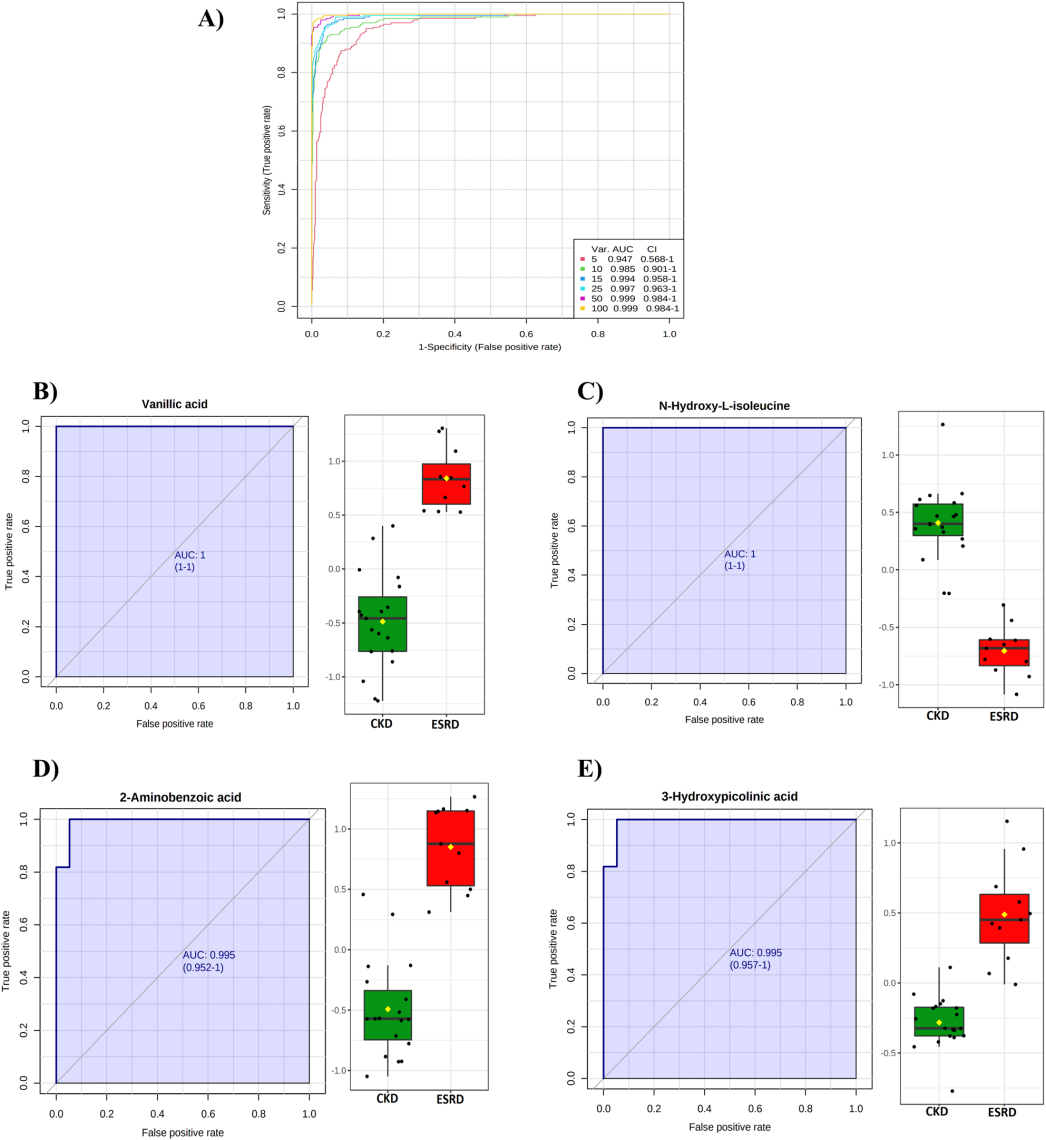
Receiver operating characteristics (ROC) curve for significantly altered metabolites between CKD and ESRD (**A**) The PLS-DA model produced a ROC with an area under the curve (AUC) of 0.985 for the top 10 variables. (**B**) Vanillic acid and (**C**) *N*-hydroxy isoleucine (both had AUC: 1) were up- and down-regulated in ESRD compared to CKD, respectively. (**D**) 2-aminobenzoic acid (AUC: 0.995) and (**E**) 3-hydroxypicolinic acid (AUC: 0.995) were up-regulated in ESRD compared to CKD.

Receiver Operating Characteristic (ROC) analysis was performed to evaluate the potential ability of these metabolites to act as biomarkers to discriminate between CKD and ESRD, Fig. 5. PLS-DA was used as a classification and feature ranking approach to creating a multivariate exploratory ROC analysis. Ten features at the exploratory ROC curve had an Area Under the Curve (AUC) value of 0.985 (95% CI), Fig. 5A. Vanillic acid and N-hydroxy-isoleucine were the highest discriminative metabolites and yielded AUC values of 1 (Fig. 5B,C). Besides, 2-aminobenzoic acid and picolinic acid were associated with a high discriminative ability with an AUC of 0.995 (Fig. 5D,E).

## Discussion

Both ESRD and CKD are major public health problems. ESRD is the final, permanent and advanced stage of CKD. In ESRD, kidney function is significantly affected leaving patients with options of either dialysis or kidney transplantation^2^. Additionally, patients with ESRD have a 100 times higher death risk for cardiovascular diseases than general population^5^. Evidence indicates that early detection of renal damage will facilitate treatment and preventive measures which can delay or even prevent progression to ESRD^26,27^. Unfortunately, current markers of kidney failure are uncertain as they are affected by several factors including age, muscle mass and patients’ medications and diet^11,39^. Previous studies reported the successful use of metabolomics to distinguish CKD at different stages and to enable the early detection of CKD even earlier than traditional clinical chemical and histopathological methods^1,23^. However, to the best of our knowledge, this approach was not used before to distinguish between CKD and its advanced state ESRD. Therefore, herein we used MS-based metabolomics approach to highlight pathways altered between ESRD and CKD and identify potential biomarkers to differentiate between the two disease states which ultimately might direct treatment and aid in slow kidney damage.

**Figure 6:**
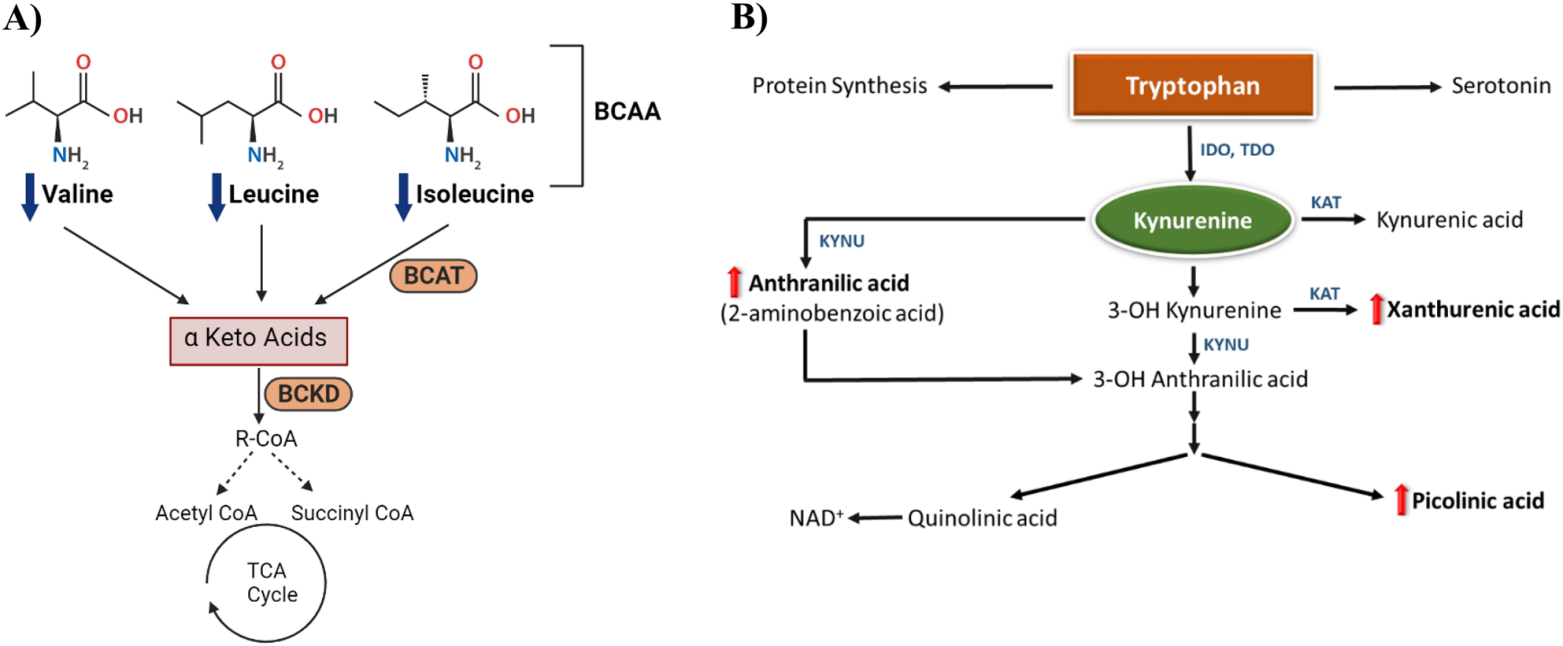
(**A**) Main steps of branched chain amino acids (BCAA) catabolism. BCAT and BCKD stand for branched-chain amino acid transaminase and branched-chain keto acid dehydrogenase, respectively. (**B**) The kynurenine pathway of tryptophan metabolism. *IDO* indoleamine 2,3-dioxygenase, *TDO* tryptophan 2,3-dioxygenase, *KAT* kynurenine aminotransaminase, *KMO* kynurenine 3-monooxygenase, *KYNU* kynureninase, *NAD*+ Nicotinamide adenine dinucleotide. Red and blue arrows indicate increase and decrease in the level of the metabolite in ESRD compared to CKD, respectively.

Among the significantly altered pathways between CKD and ESRD is aminoacyl-tRNA biosynthesis, and BCAAs metabolism. Aminoacyl-tRNA is crucial for protein biosynthesis during the pivotal process of translation while BCAA are essential amino acids with protein anabolic properties^40^. Kidney dysfunction is linked with impairment in acid secretion, increased inflammatory process and accumulation of uremic toxins^41^. Metabolic acidosis is common in patients with CKD and is worsen with the progression of the disease^42^. It has been suggested that metabolic acidosis is responsible for accelerating protein catabolism^41^. Several studies reported a decrease in the plasma level of BCAA in chronic renal failure (CRF) mainly due to metabolic acidosis, responsible for enhanced proteolysis, and the increased activity of branched-chain keto acid dehydrogenase (BCKD), a key enzyme of BCAA catabolism^40,43,44^. Patients with ESRD has a lower level of most essential amino acids, including BCAA due to decreased protein intake and hemodialysis^40^, Fig. 6A. The previous evidence is consistent with our findings where the level of BCAAs (valine, leucine, and isoleucine) was significantly lower in ESRD compared to CKD reflecting significant dysregulation of protein anabolism and catabolism in ESRD as a consequence of the progression of metabolic acidosis.

It is well known that the high inflammatory state in CKD and ESRD will result in alteration of amino acids pathway including tryptophan metabolism^45^. In the current work, activation of the tryptophan-kynurenine pathway in ESRD compared to CKD was evident by the significant decrease in the level of tryptophan and the significant elevated level of 2-aminobenzoic acid (also known as anthranilic acid), xanthurenic acid and hydroxypicolinic acid (Supplementary Table S1, Fig. 6B). As shown in Fig. 6B, the vast majority of tryptophan is metabolized by the kynurenine pathway to generate kynurenine which is further metabolized by three pathways to generate metabolites with diverse biological activity including the previously mentioned three metabolites altered herein^45^. Kynurenine derivatives play a crucial role in the modulation of several biochemical processes such as redox homeostasis, inflammation and apoptosis. Therefore, their high level might lead to cell damage and increased apoptosis rate and inflammatory responses^45–47^. The high level of toxic kynurenine metabolites enhances the generation of reactive oxygen species (ROS) and suppresses mesangial cells proliferation^48^. The previous result in mitochondrial dysfunction and high level of oxidative stress leading to aggravated kidney cell damage and an enhanced inflammatory process during the progression of CRD to ESRD^49^.

Kynurenine metabolites are excreted in the urine during the glomerular filtration process^50^. Hence, the decrease in GFR with the progression of CKD to ESRD contributes to their accumulation in the blood plasma. Of note, the level of anthranillic acid and xanthurenic acid in the blood was reported to be positively correlated with the degree of renal failure^51^. Consistent with our data many studies have shown that tryptophan-kynurenine pathway is effectively activated in CKD and reported accumulation of anthranilic acid, xanthurenic acid and picolinic acid with the progression of the CKD^45,52,53^. It is plausible that the upregulation of the tryptophan-kynurenine pathway in ESRD compared to CKD herein contributed to renal failure in these patients. It might be suggested that monitoring the level of kynurenine metabolites particularly anthranilic acid and xanthurenic acid in the blood can serve as potential biomarkers for the progression of CKD towards ESRD. Early prediction of ESRD will allow the implementation of preventive measures to prevent or delay the deterioration of kidney disease.

In the current work the level of taurine, β-amino acid involved in diverse physiologic and biologic processes in the kidney, was found to be decreased in ESRD compared to CKD. Taurine has well-recognized antioxidant properties and was found to reduce oxidant levels in diabetic nephropathy^54–56^. Additionally, it has a role in the regulation of the kidney cell cycle and apoptosis and acts as an osmolyte during the stress response^57^. The decrease in taurine level was intensified in ESRD compared to CKD, which might aid in the progression of CKD given the nephroprotective function of taurine against kidney impairment.

Expectedly and consistent with the clinical characteristics of study subjects, ESRD patients had a significantly higher level of creatinine and creatine compared to CKD (Supplementary Table S1). The elevated blood level of creatinine in CKD and ESRD has been reported^58^. Creatinine is a nitrogenous waste product formed by the breakdown of creatine in the muscle. Normally, creatinine is filtered from the blood and excreted in the urine. Therefore, its blood level is used to estimate the GFR. Our data reflects the poor ability of the kidney to filter creatinine out of the body due to the progression of CKD to ESRD resulting in serious kidney dysfunction.

The use of creatinine alone as a marker for the renal function is accompanied by a margin of inaccuracy as other factors such as diet and protein mass can result in an increase in its level. In the present study, several metabolites showed a high discriminative ability including vanillic acid, hydroxy-isoleucine and the two kynurenine derivatives 2-aminobenzoic acid and picolinic acid. The previous metabolites can serve as potential prognostic biomarkers to monitor the transition of CKD patients to ESRD, and can be used in combination with current markers to better indicate the status of kidney damage.

Vanillic acid is one of the polyphenol metabolites found in high concentrations in foods such as rice, wheat, strawberry, tea, herbs, and spices^59,60^. It exerts nephroprotective effect through its antioxidant and anti-inflammatory effects^61,62^. A recent study showed that vanillic acid could attenuate the impaired renal function in nephropathic rats by decreasing serum creatinine level and enhancing its clearance^63^. Vanillic acid is a water-soluble derivative that is rapidly excreted with urine; therefore, kidney failure can result in the retention of phenols and their metabolites in the blood^64^. The link between accumulation of vanillic acid and renal failure agrees with our results where a higher level of vanillic acid was detected in ESRD compared to CRD. Phenols derivatives such as vanillic acid accumulate differently in hemodialyzed individuals and are removed differently during hemodialysis^64^. Therefore, ESRD patients have higher concentrations of vanillic acid in blood pre-dialysis compared with direct post-dialysis.

## Conclusions

The high prevalence of CKD and its progression to ESRD are well-recognized health problems that are associated with economic implications. In this study, MS-based metabolomics approach was used for the first time to gain new insights into the perturbed biochemical pathways in ESRD compared to CKD and to identify potential prognostic biomarkers. The study illustrated that plasma metabolic profile was altered in response to renal dysfunction and the progression of CKD. Our results revealed down-regulation of BCAA metabolism in ESRD compared to CKD which might suggest the potential therapeutic role of BCAA due to their proven protein anabolic effects. However, future well-designed studies have to be performed to investigate their effectiveness in slowing down the progression of CKD. Moreover, our findings suggest that the accumulation of the kynurenine derivatives could contribute to renal damage and loss of function, resulting in kidney failure. Therefore, suppressing the kynurenine pathway of tryptophan metabolism might be a promising target to delay or even stop the progression of CKD to ESRD. A number of metabolites including hydroxy-isoleucine and 2-aminobenzoic acid and picolinic acid showed a high discriminative ability and thus may serve as potential prognostic biomarkers to monitor the progression of CKD to ESRD. The previous will improve therapeutic treatment which can delay or even prevent progression to ESRD and reduce the personal and financial burden.

One limitation of the study is the small sample size. Therefore, the potential identified biomarkers remain to be further investigated, using a targeted LC–MS approach, in a longitudinal study using a larger cohort to validate their ability to early detect kidney damage and progression of CKD, and their potential to be used in combination with current markers to provide a better indication of renal functions.

### Ethical approval

This study was performed based on a KFSHRC’ institutional research board (IRB) approved [rptocol (approval number RAC 2160027) following the Helsinki declaration (1964) and its later amendments or comparable ethical standards. Informed consent was obtained from all individual participants included in the study.

## Supplementary Information

Supplementary Information.

## Data Availability

The datasets generated during and/or analysed during the current study available from the corresponding author on reasonable request.
