# Supplementary material for: Metabolomics profiling distinctively identified end-stage renal disease patients from chronic kidney disease patients
Source: Sci Rep. 2023 Apr 15;13:6161. doi: 10.1038/s41598-023-33377-8 (PMC10105740; doi:10.1038/s41598-023-33377-8)
Supplement: Supplementary file 1 — Supplementary Information. [file 41598_2023_33377_MOESM1_ESM.pdf]

Metabolomics Profiling Distinctively Identified End-Stage Renal Disease Patients from Chronic Kidney Disease Patients

Lina A. Dahabiyeh [1], Refat M. Nimer [2], Khalid M. Sumaily [3,4], Mohamad S. Alabdaljabar [5,6], Minnie Jacob [5], Essa M. Sabi [3], Maged H. Hussein [8], Anas Abdel Rahman [5,8*]

[1] Division of Pharmaceutical Sciences, School of Pharmacy, The University of Jordan, Amman 11942, Jordan

[2] Department of Medical Laboratory Sciences, Jordan University of Science and Technology, 22110 Irbid, Jordan

[3] Clinical Biochemistry Unit, Pathology Department, College of Medicine, King Saud University, Riyadh 11461, Saudi Arabia

[4] Clinical Biochemistry Unit, Laboratory Medicine, King Saud University Medical City, King Saud University, Riyadh 11461, Saudi Arabia

[5] Metabolomics Section, Department of Clinical Genomics, Center for Genomics Medicine, King Faisal Specialist Hospital and Research Centre (KFSHRC), Riyadh 11211, Saudi Arabia;

[6] Department of Internal Medicine, Mayo Clinic, Rochester, MN, 55902, USA

[7] Department of Medicine, King Faisal Specialist Hospital and Research Centre (KFSHRC), Riyadh 11211, Saudi Arabia

[8] Department of Biochemistry and Molecular Medicine, College of Medicine, Alfaisal University, Riyadh, 11533, Saudi Arabia;

***Corresponding Author**

Anas Abdel Rahman: Metabolomics Section, Department of Clinical Genomics, Center for Genomics Medicine, King Faisal Specialist Hospital and Research Centre (KFSHRC), Riyadh 11211, Saudi Arabia; Email: aabdelrahman46@kfshrc.edu.sa

List of Supplementary Figures and Table

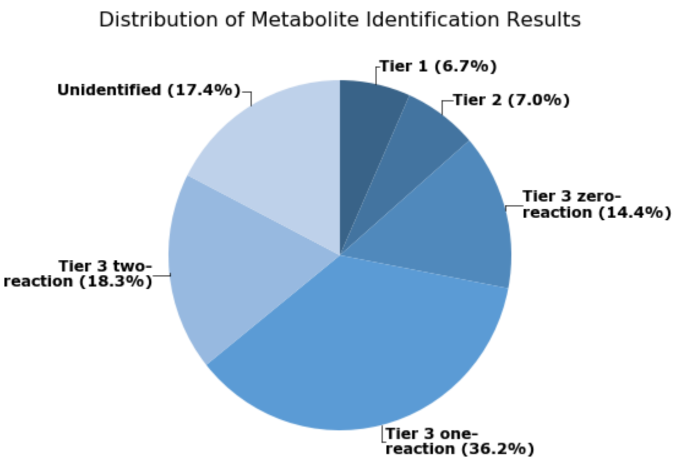

Fig. S1 Distribution of the results for metabolites identification using Tier 3 approach

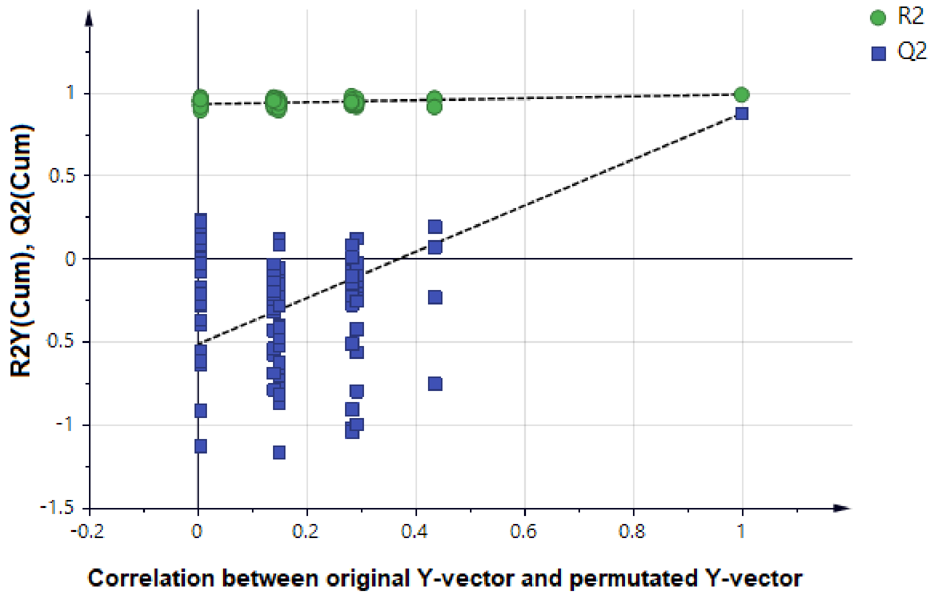

Fig. S2 Permutation test for validating the OPLS-DA model generated from the binary comparison between CKD and ESRD. Q^2 (blue squares) and R^2Y (green circles). Values from the permuted analysis models (left- side) should be less than the initial generated model values (right- corner)

ESRD: end-stage renal disease; CKD: chronic kidney disease

Table S1 Significantly altered metabolites in end-stage renal disease compared to chronic kidney disease with VIP >1 (in multivariate analysis and false discovery rate (FDR) < 0.05 (in univariate analysis) Metabolites with VIP >1 in multivariate analysis

No.	Metabolite	FDR	VIP	FC (ESRD/CKD)
1	(R)-1-Aminopropan-2-ol	8E-05	1.81	2.36
2	(S)-4-Amino-5-oxopentanoic acid	2E-03	1.55	0.50
3	(S)-4-Hydroxymandelonitrile	1E-02	1.25	2.92
4	(S)-5-Amino-3-oxohexanoic acid	4E-02	1.08	1.66
5	1,2,3-Trihydroxybenzene	1E-03	1.60	5.08
6	1,3-Diaminopropane	2E-03	1.54	1.72
7	1-Aminocyclopropane-1-carboxylic acid	3E-02	1.19	0.74
8	2,4-Diaminotoluene	3E-02	1.15	0.54
9	2,5-Dihydroxybenzoic acid	5E-04	1.66	3.31
10	2',6'-Dihydroxy-4'-methoxyacetophenone	4E-04	1.68	5.03
11	2-Amino-2-methyl-1,3-propanediol	1E-02	1.32	0.64
12	2-Amino-5-oxohexanoic acid	3E-02	1.13	2.23
13	2-Aminobenzoic acid	2E-02	1.21	2.74
14	2-Aminomuconic acid	3E-03	1.52	2.64
15	2-Carboxy-2,3-dihydro-5,6-dihydroxyindole	1E-06	2.09	10.46
16	2-Hydroxy-cis-hex-2,4-dienoic acid	5E-05	1.87	3.33
17	2-Hydroxypyridine	2E-02	1.27	1.65
18	2-Phenylglycine	6E-03	1.44	2.37
20	3-(2,3-Dihydroxyphenyl) propanoic acid	1E-02	1.30	1.88
21	3-(3,4-Dihydroxyphenyl)lactic acid	1E-07	2.16	19.47
22	3,4-Dihydroxyphenylpropanoic acid	2E-04	1.72	5.69
23	3-Chlorocatechol	1E-02	1.29	0.71
24	3-Cresotinic acid	5E-03	1.44	2.55
25	3-Hydroxyanthranilic acid	2E-03	1.51	3.23
26	3-Hydroxyphenylacetic acid	3E-03	1.52	1.80
27	3-Hydroxypicolinic acid	1E-06	2.05	2.35
28	4-Aminobenzoic acid	7E-03	1.36	0.76
29	4-Aminobutyraldehyde	1E-02	1.35	0.63
30	4-Hydroxy-3-methylbenzoic acid	4E-05	1.87	2.18
31	4-Hydroxyaniline - 2 tags	1E-02	1.35	0.62
32	4-Hydroxybenzoic acid	3E-03	1.50	1.91
33	4-Hydroxy-L-tryptophan	5E-03	1.43	1.67
34	4-Hydroxyphenylacetylglutamic acid	4E-06	1.99	5.36
35	4-Oxoproline	2E-02	1.21	0.70
36	5-Aminopentanal	1E-06	2.09	0.67

No.	Metabolite	FDR	VIP	FC (ESRD/CKD)
37	5-Hydroxyindoleacetic acid	2E-05	1.92	3.87
38	5-Hydroxymethyluracil	2E-02	1.22	0.52
39	5-L-Glutamyl-taurine	4E-02	1.17	0.47
40	5-Methoxysalicylic acid	3E-03	1.45	2.63
41	7,8-Diaminononanoic acid	3E-03	1.52	1.96
42	7,8-Dihydroxanthopterin	5E-04	1.67	2.56
43	Adenosine monophosphate	4E-02	1.15	0.53
44	Alanine	1E-02	1.34	0.66
45	Alanyl-Glutamic acid	4E-05	1.87	0.29
46	Alloisoleucine	2E-04	1.72	0.62
47	Alpha-aminobutyric acid	2E-03	1.51	0.45
48	Aminoacrylic acid	4E-04	1.70	0.65
49	Ascorbic acid	1E-06	2.06	5.02
50	Asparaginyl-Aspartic acid	9E-03	1.34	2.09
51	Asparaginyl-Glutamic acid	1E-02	1.27	1.65
52	Aspartyl-Arginine	3E-03	1.53	2.26
53	Aspartyl-Aspartate	7E-03	1.39	1.53
54	Aspartyl-Glutamate	1E-03	1.58	1.43
55	Aspartyl-Phenylalanine	7E-03	1.38	2.50
56	Aspartyl-Proline	4E-03	1.42	1.48
57	Beta-Alanine	4E-02	1.17	0.63
58	Beta-Guanidinopropionic acid	3E-06	2.02	2.03
59	Caffeyl alcohol	1E-02	1.28	1.57
60	Calystegin	4E-06	1.99	2.99
61	Creatine	1E-05	1.93	2.72
62	Creatinine	1E-06	2.08	3.39
63	Cysteinyldopa	1E-06	2.07	3.50
64	Dihydrocapsaicin	4E-03	1.46	0.74
65	D-Lysopine	1E-04	1.79	2.55
66	gamma-Glutamylalanine	3E-02	1.22	0.55
67	Glutaminyl-Aspartic acid	1E-04	1.80	2.37
68	Glutamyl-Valine	4E-04	1.67	2.06
69	Glycyl-Aspartate	2E-04	1.73	2.33
70	Glycyl-Proline	1E-02	1.30	2.24
71	Histidine	3E-03	1.52	0.71
72	Histidinyl-Aspartate	6E-04	1.65	2.84
73	Histidinyl-Glutamate	3E-04	1.72	0.47
74	Histidinyl-Tryptophan	3E-02	1.15	2.08
75	Homoarginine	7E-03	1.37	0.59

No.	Metabolite	FDR	VIP	FC (ESRD/CKD)
76	Homocitrulline	1E-02	1.26	1.79
77	Homogentisic acid	1E-03	1.58	2.50
78	Homomethionine	4E-02	1.14	1.82
79	Homoserine	3E-02	1.21	0.40
80	Homovanillic acid	2E-04	1.72	3.24
81	Hydroquinone	2E-02	1.26	1.74
82	Hypoglycin B	4E-04	1.68	2.12
83	Isoleucine	3E-04	1.72	0.62
84	Isoleucyl-Aspartate	1E-03	1.63	0.44
85	1-Aminocyclopropane-1-carboxylic acid	1E-02	1.29	0.59
86	2-Amino-2-methyl-1,3-propanediol	1E-02	1.33	0.64
87	2-Aminobenzoic acid	1E-07	2.19	9.20
88	2-Hydroxy-cis-hex-2,4-dienoic acid	2E-04	1.76	0.63
89	3-Sulfocatechol	8E-06	1.96	7.68
90	4-Aminobutyraldehyde	1E-02	1.32	0.64
91	4-Hydroxybenzoic acid	7E-05	1.83	3.18
92	5-Aminopentanal	1E-04	1.82	0.75
93	5-Hydroxyindoleacetic acid	1E-03	1.63	2.40
94	7,8-Dihydroxanthopterin	3E-03	1.54	2.58
95	Alanine	1E-02	1.34	0.66
96	Alpha-aminobutyric acid	1E-02	1.31	0.75
97	Asparaginyl-Aspartic acid	1E-02	1.33	2.07
98	Caffeyl alcohol	6E-03	1.38	1.58
99	Creatinine	3E-06	2.02	3.16
100	Glutamine	2E-05	2.09	2.92
101	Glycyl-Proline	1E-02	1.32	2.26
102	Homoserine	7E-04	1.63	1.66
103	Hydroxyphenyllactici acid	8E-06	1.96	5.07
104	Isoglutamine	2E-04	1.78	0.58
105	Leucyl-Aspartate	1E-04	1.80	3.90
106	Leucyl-Glutamate	2E-03	1.57	0.54
107	Lysine	3E-05	1.86	0.66
108	Phenylalanyl-Serine	2E-06	1.99	3.40
109	Serine	2E-02	1.31	0.76
110	Tabtoxinine-beta-lactam	2E-02	1.25	2.79
111	IsoValine	2E-05	1.91	0.56
112	Valyl-Aspartate	1E-06	2.06	4.10
113	5-Aminopentanal	1E-06	2.08	0.65
114	4-Aminobutyraldehyde	1E-02	1.34	0.63

No.	Metabolite	FDR	VIP	FC (ESRD/CKD)
115	5-Hydroxyindoleacetic acid	1E-02	1.29	0.55
116	Alanine	1E-02	1.35	0.78
117	Hydroxyphenyllactici acid	5E-05	1.84	6.80
118	Lysine	1E-04	1.80	0.66
119	Serine	2E-03	1.54	0.71
120	Valine	2E-05	1.90	0.57
121	4-Aminobutyraldehyde	1E-02	1.36	0.62
122	5-Aminopentanal	2E-06	2.04	0.67
123	5-Hydroxyindoleacetic acid	2E-02	1.28	0.56
124	Proline	4E-02	1.12	0.80
125	4-Aminobutyraldehyde	1E-02	1.33	0.65
126	5-Aminopentanal	2E-06	2.06	0.67
127	sarcosine	2E-02	1.25	0.77
128	L-2-Aminoadipate 6-semialdehyde/Allysine	3E-02	1.11	1.48
129	L-3-Aminodihydro-2(3H)-furanone	4E-04	1.68	2.24
130	L-Cystathionine - 2 tags/Allocystathionine	3E-04	1.72	2.29
131	Leucine	2E-04	1.72	0.62
132	Leucyl-Aspartate	9E-05	1.84	3.98
133	Leucyl-Glutamate	3E-03	1.55	0.55
134	L-gamma-Glutamyl-(3R)-L-beta-ethynylserine	1E-04	1.82	3.53
135	L-Norleucine	1E-03	1.59	0.65
136	L-Pipecolic acid	5E-02	1.18	0.41
137	Lysine	1E-04	1.79	0.66
138	Lysyl-Proline	2E-04	1.71	0.54
139	Methionyl-Aspartate	2E-03	1.55	3.76
140	Methylguanidine	1E-05	1.94	3.66
141	N2-Succinyl-L-ornithine	9E-03	1.36	1.56
142	N6-Acetyl-LL-2,6-diaminoheptanedioic acid	5E-03	1.41	1.60
143	N-Alpha-acetyllysine	1E-04	1.79	2.47
144	Neuraminic acid	5E-05	1.84	2.32
145	N-Hydroxy-L-isoleucine	5E-08	2.21	0.19
146	N-Hydroxy-L-valine	7E-05	1.85	0.41
147	Ophthalmic acid	4E-04	1.67	3.78
148	Ornithine	1E-02	1.34	0.67
149	Ortho-Hydroxyphenylacetic acid	1E-04	1.80	2.08
150	Phenylalanyl-Aspartate	2E-06	2.04	4.25
151	Porphobilinogen	4E-02	1.13	0.66
152	Proline	5E-03	1.41	0.75
153	Prolyl-Alanine	6E-03	1.40	0.62

No.	Metabolite	FDR	VIP	FC (ESRD/CKD)
154	Prolyl-Aspartate	7E-05	1.81	1.80
155	Prolyl-Histidine	3E-03	1.56	0.66
156	Prolyl-Leucine	2E-03	1.56	0.59
157	Prolyl-Lysine	7E-04	1.63	0.64
158	Prolyl-Serine	4E-02	1.17	0.70
159	Prolyl-Tyrosine	2E-02	1.20	0.68
160	Prolyl-Valine	1E-04	1.81	0.55
161	Resorcinol	2E-02	1.23	1.78
162	S-Adenosylhomocysteine	4E-05	1.90	3.14
163	Serine	6E-04	1.67	0.65
164	Seryl-Aspartic acid	7E-05	1.83	3.10
165	Syringic acid	4E-04	1.66	2.38
166	Taurine	4E-02	1.16	0.53
167	Threonine	5E-02	1.12	0.72
168	Threoninyl-Aspartate	5E-03	1.39	1.58
169	Threoninyl-Glutamate	5E-03	1.49	0.60
170	trans-Ferulic acid	3E-03	1.51	2.34
171	Tryptophan	9E-03	1.38	0.60
172	Tyrosyl-Aspartate	2E-05	1.94	3.85
173	Valine	2E-05	1.90	0.57
174	Valyl-Aspartate	2E-04	1.74	2.02
175	Valyl-Phenylalanine	9E-03	1.28	0.52
176	Vanillic acid	2E-07	2.17	8.46
177	Xanthurenic acid	2E-04	1.79	2.80
178	Xanthurenic acid	2E-04	1.79	2.99
